# Managing Predicted Post-Orthognathic Surgical Defects Using Combined Digital Software: A Case Report

**DOI:** 10.3390/healthcare11091219

**Published:** 2023-04-25

**Authors:** Neculai Onică, Cezara Andreea Onică, Monica Tatarciuc, Elena-Raluca Baciu, Georgiana-Lena Vlasie, Mihai Ciofu, Mihail Balan, Gabriela Luminița Gelețu

**Affiliations:** 1 Specialist Oral and Maxillofacial Surgery, Private Practice, 700612 Iasi, Romania; nicuonica@yahoo.com; 2 Specialist Oral Surgery, Private Practice, 700612 Iasi, Romania; dr.cezaraonica@yahoo.com; 3 Department of Implantology, Removable Dentures, Dental Technology, Faculty of Dental Medicine, University of Medicine and Pharmacy, “Grigore T. Popa”, 700115 Iasi, Romania; 4 Specialist Orthodontics, Private Practice, 700612 Iasi, Romania; georgiana-lena-vlasie@email.umfiasi.ro; 5 Department of Surgery, Faculty of Dental Medicine, University of Medicine and Pharmacy “Grigore T. Popa”, 700115 Iasi, Romania; mihai.ciofu@umfiasi.ro (M.C.); mihail.balan@umfiasi.ro (M.B.); gabriela.geletu@umfiasi.ro (G.L.G.)

**Keywords:** orthognathic surgery, genioplasty, computer-assisted designs, computer-aided manufacturing

## Abstract

For facial abnormalities, recent developments in virtual surgical planning (VSP) and the virtual design of surgical splints are accessible. Software companies have worked closely with surgical teams for accurate outcomes, but they are only as reliable as the data provided to them. The current case’s aim was to show a fully digitized workflow using a combination of three digital software to correct predicted post–upward sliding genioplasty defects. To reach our goal, we presented a 28-year-old man with long-face syndrome for orthodontic treatment. Before orthognathic surgery, a clinical and paraclinical examination was performed. For a virtual surgical plan, we used the dedicated surgical planning software NemoFab (Nemotec, Madrid, Spain) and Autodesk MeshMixer (Autodesk Inc., San Rafael, CA, USA). To create the design of the digital guides, DentalCAD 3.0 Galway (exocad GmbH, Darmstadt, Germany) and Autodesk MeshMixer (Autodesk Inc., San Rafael, CA, USA) were used. The patient had undergone bilateral sagittal split osteotomy in addition to Le Fort 1 osteotomy and genioplasty, followed by mandible base recontouring ostectomy. Stable fixation was used for each osteotomy. Based on our case, the current orthognathic surgery planning software was not able to perform all the necessary operations autonomously; therefore, future updates are eagerly awaited.

## 1. Introduction

For the last 40 years, model block surgery, alongside traditional two-dimensional (2D) cephalometric radiographs, has been a valuable tool in planning orthognathic surgery treatment. 

Initially, surgeons and orthodontists primarily focused on occlusion and mastication function. However, over time, there has been growing concern about improving other functions, such as breathing and facial aesthetics. Despite their significance in planning and the final outcome, they did not give much information about the osteotomy sites after repositioning the bones to the desired position. An untrained eye could easily overlook unwanted gaps, interferences, and steps. 

Computer-aided design and manufacturing (CAD/CAM) has revolutionized craniomaxillofacial surgery by improving the planning of osteotomies, the fabrication of splints, the development of osteotomy and ostectomy guides, the stereolithography of bones, and even custom osteosynthesis plates [1,2,3].

Digital planning is a specific tool that is gaining importance every day. Surgeries that were once deemed impossible can now be performed regularly worldwide thanks to digital advancements. 

Virtual surgery planning (VSP) is a computer-assisted process that uses cone beam computed tomography (CBCT) images of a patient’s face and jaw to create a virtual model of the patient. This virtual model can then be used to simulate orthognathic surgical procedures, allowing the surgeon to evaluate the feasibility of different treatment plans and provide the patient with a more precise description of the possible outcomes. Thanks to these developments, the communication and understanding between patients and medical teams have been improved [4].

In the current literature, the most listed commercially available VSP software is Dolphin Imaging (Dolphin Imaging & Management Solutions, Los Angeles, CA, USA), Maxilim (Medicim NV, Mechelen, Belgium), SimPlant OMS (Materialise NV, Leuven, Belgium), and Mimics (Materialise NV, Leuven, Belgium) [5,6]. A large number of surgeons, each with their own unique approaches, puts substantial pressure on software companies to address both general and individual needs.

One of the biggest benefits of VSP is the improved visualization of craniofacial deformities, such as occlusal canting and asymmetries [7,8,9]. In addition, the surgeon can have an accurate image of the relationship between bony fragments concerning wanted/unwanted gaps, interferences, and steps along the position of the central upper incisor, the inclination of the occlusal plane, cant, and yaw. VSP is believed to be less time-consuming [10], less expensive, and with lower complication incidence than conventional surgery planning, based on 2D cephalometric and patient images, face-bow, articulators, and mounted dental models [8,11,12]. Despite numerous benefits, there is limited information available on the challenges of this method. 

The natural head position (NHP), also known as natural head posture, was first defined in 1862 as ‘when man is standing and his visual axis is horizontal, he is in the natural position’ [13,14]. The true horizontal line (THL) and true vertical line parameters (TVL) are established by using landmarks on the patient’s face, which ensure that surgical corrections made to the jaw and teeth align properly with the rest of the face [15]. Deviations from the TVL can result in an unbalanced and aesthetically unsatisfactory result. In order to achieve the desired outcomes, it is crucial for the surgeon to accurately establish the true vertical line and maintain its integrity throughout the surgical procedure. The use of NHP (combined with TVL and THL) is preferred due to its efficiency, stability, and improved effectiveness in the analysis [16].

Currently available VSP software is based on soft tissue cephalometric analysis (STCA). According to Arnett et al. [17], the soft tissue profile is an important indicator for obtaining facial harmony, tooth placement, and occlusal correction. 

This paper aims to present a fully digitized workflow using a combination of three digital software to correct the discontinuities in the mandible body obtained by an upward sliding genioplasty. 

## 2. Case Report

A 28-year-old man with long face syndrome, under orthodontic treatment for 2 years, was referred to our dental office for orthognathic surgery (OS) intervention. He had no systemic diseases or family history of significant issues.

His main complaints were excessive tooth exposure, a gummy smile, and a long and retruded chin. 

Before OS, a clinical examination, CBCT, and intraoral scans of the dental arches and occlusion were performed.

The CBCT images were acquired using a DentriMax CBCT (DentriMax, HDX Will, Seoul, Republic of Korea) with the following parameters 90 kV, X-ray tube current = 10 mA, Field of view (FOV) 18/16.5, focal spot = 0.5 mm, Voxel size = 0.10. the CBCT scans were acquired in the natural head position (NHP) in an upright position. The condyles were sited in centric relation (CR), and the preoperative occlusion in CR was fixed with a wax-byte at the preoperative CBCT scanning; however, without the wax-byte at the postoperative CBCT scanning acquisition. The CBCT data were exported in the digital imaging and communications in medicine (DICOM) format.

**Figure 1:**
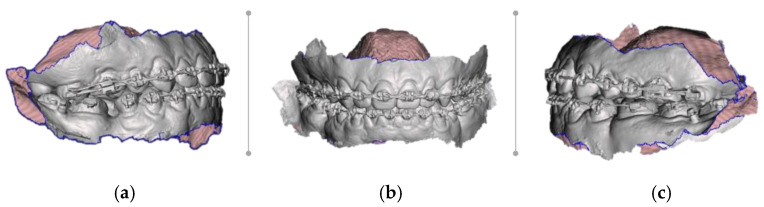
Pre-surgical paraclinical intraoral scans: (**a**) right lateral view; (**b**) frontal view; and (**c**) left lateral view.

Intraoral scans were performed with Medit i700 (MEDIT corp. 8, Seoul, Republic of Korea), with the following parameters: 3D motion video technology/ 3D full-color streaming capture, scanning frame up 70 FPS, tip size 22.2 × 15.9 mm, 45-degree mirror angle, scan at 15/13 mm, and recorded images were saved as standard tessellation (.STL) files (Figure 1a–c).

The clinical examination was confirmed by the cephalometric analysis of a skeletal class III patient (Table 1 and Table 2).

**Figure 2:**
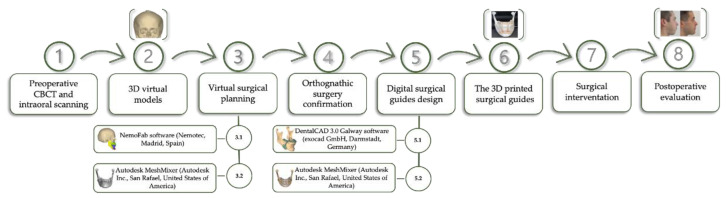
The orthognathic surgery workflow.

In Figure 2, an overview of the proposed framework is illustrated.

Consent was obtained after the patient was informed of the diagnosis, the prognosis with and without therapy, the specific treatment steps, and the benefits of the procedures, including specific risks and possible adverse effects.

### 2.1. Virtual Surgical Plan

**Figure 3:**
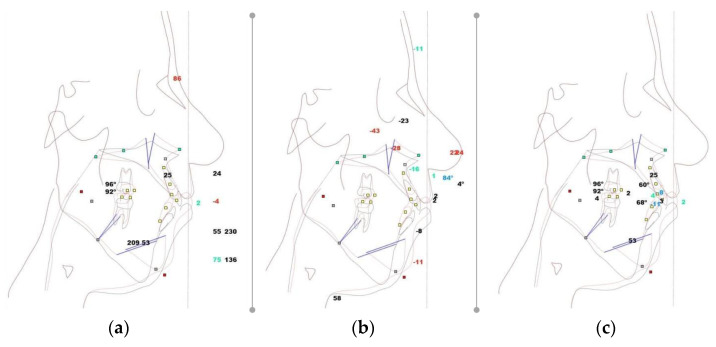
Cephalometric analyses using NemoFab (Nemotec, Madrid, Spain): (**a**) Dentoskeletal factors; (**b**) Projections; (**c**) Heights.

All obtained data were first imported into NemoFab surgical planning software (Nemotec, Madrid, Spain) before being analyzed (Figure 3a–c).

**Figure 4:**
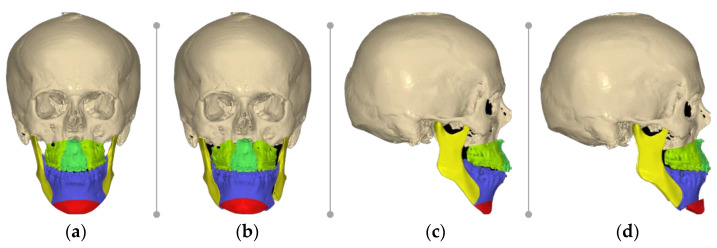
Pre-surgical computer-assisted virtual simulation using NemoFab (Nemotec, Madrid, Spain): (**a**) frontal view of patient skull; (**b**) frontal view of planned final reposition; (**c**) lateral view of patient skull; and (**d**) lateral view of planned final reposition.

We first opted for an upward sliding genioplasty rather than a reduction in the anterior mandible because the latter would not achieve the proper advancement needed, the mandible angle degree would not be decreased, and would not preserve bony contact (Figure 4a–d).

By selecting this solution, the anterior mandible height and the proper position of the Pogonion (Pg) to the TVL were both resolved, but this resulted in a 7.5 mm step between the posterior wing of the chin and the body of the mandible, which was detrimental to a continuous base of the mandible.

**Figure 5:**
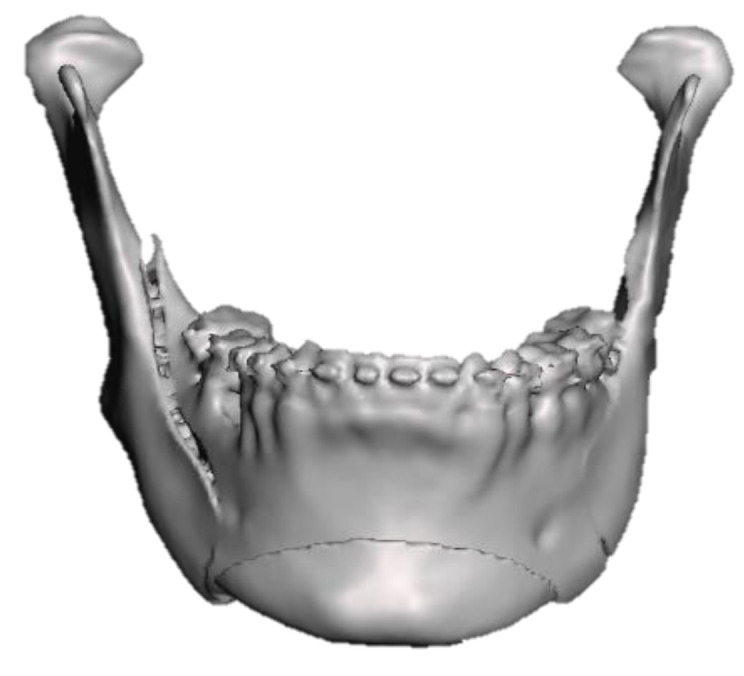
The combination of all the mandible fragments into a single .STL file using Autodesk MeshMixer (Autodesk Inc., San Rafael, CA, USA).

Since the NemoFab surgical planning software did not allow us to design a new set of osteotomies, it was necessary to export the .STL files of the mandible in its final position (both condylar bearing fragments, body of mandible, and upward sliding genioplasty) into software capable of performing Boolean operations (the union). For this purpose, Autodesk MeshMixer (Autodesk Inc., San Rafael, CA, USA) was used (Figure 5).

### 2.2. Digital Surgical Guides Design

**Figure 6:**
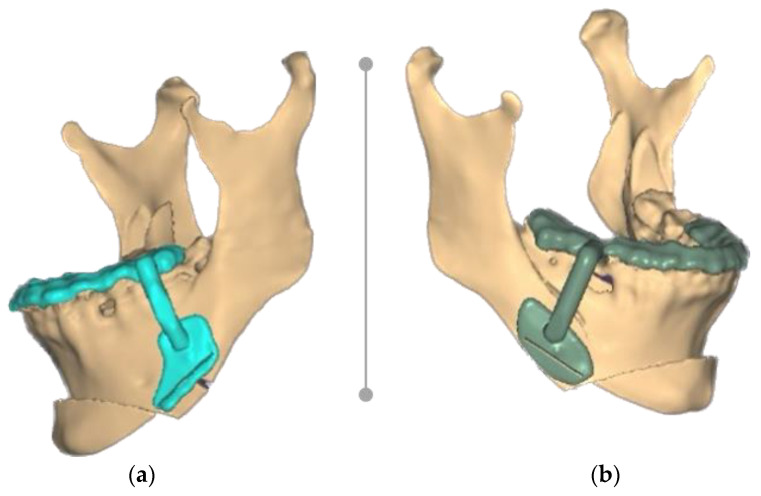
Virtual images of the designed guides using DentalCAD 3.0 Galway (exocad GmbH, Darmstadt, Germany): (**a**) left lateral view; and (**b**) right lateral view.

Following this step, a single unified .STL file was exported into DentalCAD 3.0 Galway software (exocad GmbH, Darmstadt, Germany) to create three digital surgical guides: one mental and two laterals (Figure 6a,b). Although NemoFab included a guided designing option, it could only be used after the bones were initially positioned with the osteotomy design.

**Figure 7:**
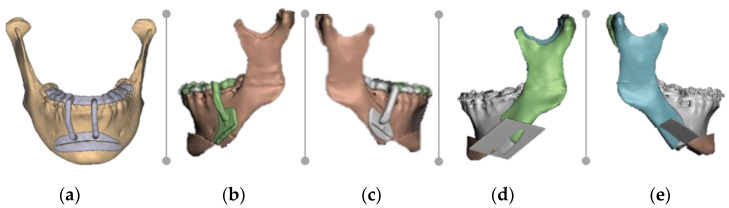
Virtual images of the designed guides and the reduction plane using MeshMixer (Autodesk Inc., San Rafael, CA, USA): (**a**) frontal view of anterior guide; (**b**) left lateral view of lateral reduction guide; (**c**) right lateral view of lateral reduction guide; (**d**) left lateral view of reduction plane; and (**e**) right lateral view of reduction plane.

Since DentalCAD 3.0 Galway (exocad GmbH, Darmstadt, Germany) was also deficient in Boolean operations, the .STL files of the guides and the mandible with the fragments in their final positions were then exported to Meshmixer (Autodesk Inc., San Rafael, CA, USA) to complete the final process (Figure 7a–e).

In our case, five digital guides were created: an intermediate splint, a palatal splint, lateral reduction guides left and right, and an anterior guide (mental guide).

### 2.3. Digital Guides Fabrication

**Figure 8:**
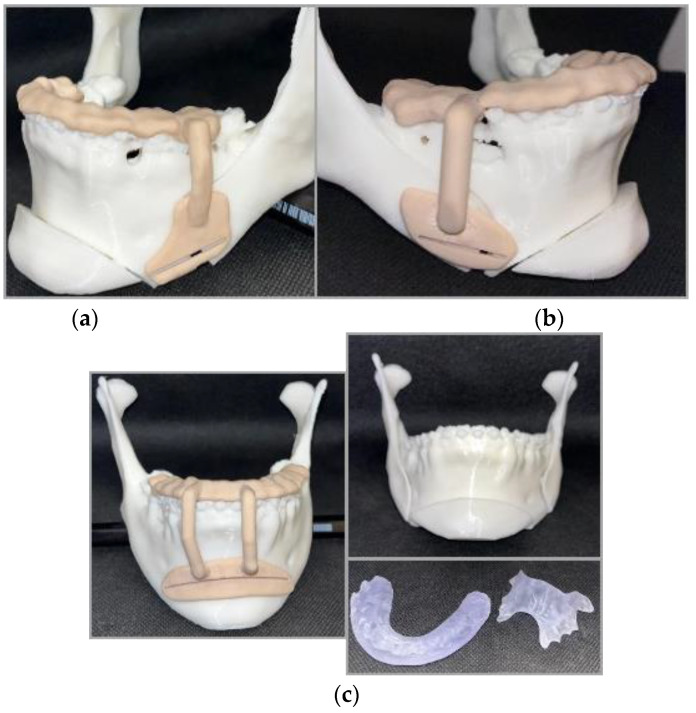
Three-dimensional printed digital guides and splints: (**a**) left lateral view of lateral reduction guide; (**b**) right lateral view of lateral reduction guide; and (**c**) frontal view of mental guide and the intermediate and palatal splints.

The surgical guides were fabricated from acrylic resin (Phrozen Water-Washable Dental Model 3D Printer Resin, Phrozen Technology, Hsinchu, Taiwan) using an ASIGA 3D MAX UV printer (ASIGA, Alexandria, NSW, Australia) before they were then sterilized for surgery (Figure 8a–c).

Sterilizing by immersion for 25 min in peracetic acid (Gigasept PAA, Schülke & Mayr GmbH, Norderstedt, Germany) was performed prior to surgery.

### 2.4. Surgical Intervention

Blood tests were in the normal range.

The patient had surgery while under general anesthesia and nasotracheal intubation. In order to reduce bleeding and facilitate tissue dissection, a vasoconstrictor solution (6 vials of 1.7 mL of articaine, 1 vial of epinephrine, 1 vial of tranexamic acid, and 100 mL of saline) was used locally. 

Bilateral sagittal split osteotomy (BSSO) was performed using an intermediate splint. The fragments were fixed with single 4-hole straight plates (I plate, Medicon eG, Tuttlingen, Germany), bilaterally 2/6 mm monocortical screws (Medicon eG, Tuttlingen, Germany), and two bicortical screws (2/13 mm, Medicon eG, Tuttlingen, Germany) inserted on either side of the osteotomy.

Mental osteotomy followed, upward sliding it into the new position to correct the anterior mandible height and securing the new position by using a prebent rectangular 5 holes plate and monocortical screws (2/6 mm, Medicon eG, Tuttlingen, Germany), which led to a 7 mm advancement.

**Figure 9:**
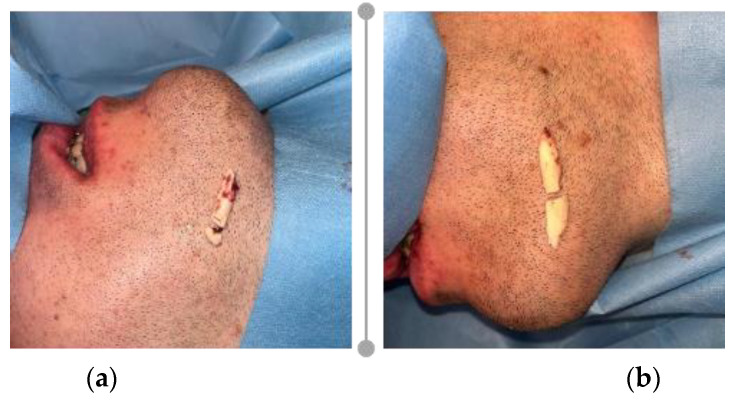
Resulted bone fragments from mandible body marginal base reduction: (**a**) right lateral view; and (**b**) left lateral view.

The prior designed reduction guides were placed in the designated position using the same access for BSSO with a slight anterior reflection of the periosteum until and beneath the mental foramen. To facilitate the placement of the guide and to retain visual control of the osteotomy, mental and BSSO accesses were united by a tunneling reflection of the periosteum beneath the mental nerve (Figure 9a,b). Mandible osteotomy was performed using a piezoelectric instrument (Woodpecker Ultrasurgery, Guilin Woodpecker Medical Instrument Co., LTD., Guilin, China) with a properly angled tip (US1L-Left Angle 90 degrees Micro-Saw 0.5 mm, Guilin Woodpecker Medical Instrument Co., LTD., Guilin, China).

Maxillary excess was treated with a multisegmental Le Fort I osteotomy, which involved septoplasty and inferior turbinate reduction, followed by a 5 mm clinical vertical repositioning to return the maxilla to its correct occlusal position. There was no final splint used. For the fixation, two double plates were applied, each with seven monocortical 2/5 mm screws (Medicon eG, Tuttlingen, Germany). Again, osteotomy gaps were filled using a ground homologous bone graft collected from the impaction sites.

**Figure 10:**
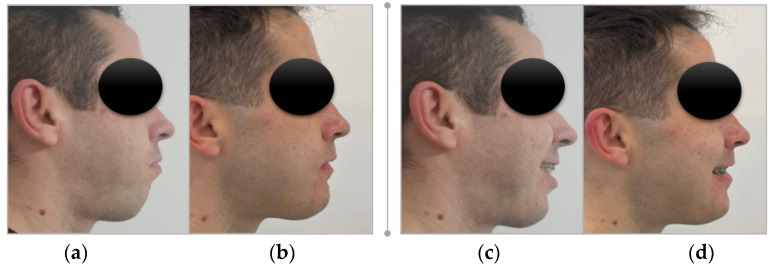
The extraoral images of patient profile: (**a**,**c**) preoperative; and (**b**,**d**) 7 days postoperative.

**Figure 11:**
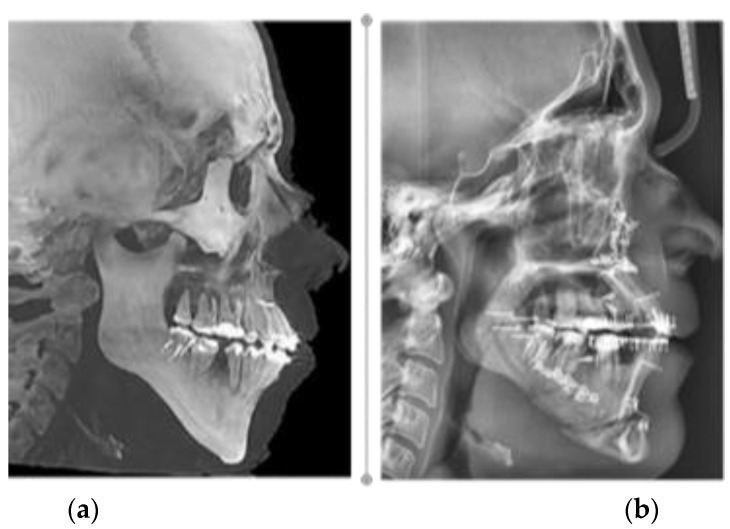
The profile teleradiography images: (**a**) preoperative; and (**b**) 3 weeks postoperative.

From the first week after orthognathic surgery, there was an aesthetic significant improvement in facial appearance (Figure 10a–d and Figure 11a,b).

Postoperative treatment included 7 days of intravenous antibiotic therapy and regular pain medications. An ice pack was used intermittently in the first 72 h following the intervention.

During the first three days post-surgery, the surgeon cleaned the patient and instructed him on how to maintain proper oral hygiene using a soft toothbrush and rinsing with a chlorhexidine solution for ten days. The patient was advised to follow a liquid diet for a month and was given elastic therapy for 40 days, after which he started post-surgical orthodontic treatment.

### 2.5. Cephalometric Planes

Using the true vertical line in a natural head position state as a landmark for facial aesthetics, the modified parameters are described in Table 3.

The mandibular angle degree significantly decreased from 136.49° to 128.7°. The width of the mandible (Go-Go) increased from 90.5 mm (pre-surgery) to 96.2 mm (post-surgery).

## 3. Discussion

Long faces with maxillary or/and mandibular hyperplasia with or without asymmetry require a special interdisciplinary approach. Digital planning becomes increasingly important in these situations. The confection and use of different splints and surgical guides ensure the high predictability of surgical outcomes. 

Occlusion or oral function correction is the most frequent reason for orthognathic surgery, followed by improvements to aesthetic and psychosocial functions. From the patient’s point of view, facial appearance is one of the primary motives for accepting surgical interventions [18]. A descriptive quasi-experimental study conducted by Rezaei et al. [19] on 112 Persian adult patients with class III skeletal malocclusion before and after OS revealed that surgery interventions enhanced the individuals’ quality of life, as well as their contentment, self-confidence, and oral function. The current case report supports such findings since the patient was satisfied with the aesthetic and functional outcomes.

The treatment plan varied from one team to another, with OS indicated either before or after orthodontic treatment. In the recent literature, the term” surgery first” is described with potential benefits in shortening treatment time and improving facial aesthetics from begging [20,21]. OS was proposed after the patient had undergone orthodontic therapy for two years.

This therapeutic approach consisted of two stages. The first stage involved an orthodontic strategy comprising decompensation and alignment. The maxillary arch was maintained and unchanged while correcting the mandibular Spee and Wilson curves. The second stage involved a surgical strategy, which encompassed maxillo-mandibular repositioning in all three spatial axes, along with the counterclockwise (CCW) rotation of the occlusal plane, in this case, of a hyper-divergent mandibular pattern. Our decision was influenced by the desire to establish an aesthetic profile connected to TVL and NHP aesthetic planes.

The current VSP software exhibits constrained versatility and limited applicability when used for the analysis of osteotomies executed in orthognathic surgery [22]. In our case, NemoFab (Nemotec, Madrid, Spain) did not allow us to perform osteotomies after virtual surgical planning.

Bone reduction in this region was indicated as a viable treatment for the bottom border of the mandible’s aesthetic imbalance; in order to make the process more precise, we chose the digital method. NemoFab surgical planning software (Nemotec, Madrid, Spain) predicted that a major step would be present after upward sliding genioplasty. 

The patient had undergone BSSO in addition to Le Fort 1 osteotomy and genioplasty followed by mandible base recontouring ostectomy. Stable fixation was used for each osteotomy. 

Hernández-Alfaro [23] stated that the surgical management of Long Face Syndrome could involve the following methods, either alone or in combination: maxillary impaction, vertical chin reduction, and the counterclockwise rotation of the occlusal plane. In some circumstances, another procedure, guided mandible base ostectomy, could be added to achieve a straight line and a continuous mandible base.

By impacting the anterior part of the maxilla by 5 mm, the position of the chin was moved 3 mm superior, and the mandible was advanced by 1 mm. The same findings were reported by Jayakumar et al. [24] in a study on 45 patients with vertical maxillary excess. The authors observed that a superior maxillary impaction of 1 mm generated a chin movement of 0.6 mm vertically and 0.2 mm sagittally.

The 9-degree flattening of the occlusal plane translated into an advancement of 8 mm and an improvement of 4 mm, and the upward sliding genioplasty resulted in an advancement of 7 mm and an improvement of 5 mm.

The potential for recognizing consequences, including mental nerve injury and step deformity, is mentioned in the specialized literature [25,26].

The usage of only two dedicated software programs could be considered a limitation of the current case report. In light of this constraint, a comparative analysis of clinical case resolutions using commercially available software is required.

## 4. Conclusions

Rehabilitation standards in dentistry have evolved and improved as a result of new multidisciplinary concepts and more complex perspectives on additional interventions that may help patients to restore function, health, and aesthetics.

The outcomes of combining these software results proved satisfactory for both the medical team and the patient. The use of cutting guides reduced the duration of the surgery and increased the precision of mandible base ostectomy, resulting in a continuous and improved mandible base ostectomy. 

In our case, current orthognathic surgery planning software was unable to perform all the necessary operations autonomously; thus, future updates should make single software applications possible, reducing the amount of time required for learning the software and the amount of money required for multiple licenses.

## Figures and Tables

**Table 1:** Soft tissue cephalometric analysis (STCA)—projections using NemoFab (Nemotec, Madrid, Spain).

STCA Projections					
Cephalometric Variables		Normal Range	Patient	Retruded	Protruded
High midface Projection	Glabella	−9.5 to 4.5	−11	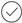	-
	Orbital rims	−21 to −15	−22.9	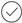	-
	Cheekbones	−27 to −17	−43.3	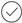	-
	Subpupil	−18 to −12	−28.5	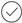	-
Maxillary Projection					
	Nasal base	−14 to −10	−15.6	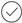	-
	Nasal Tip	13.2 to 18.2	24.1	-	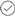 Large
Soft tissue Projection	Upper lip angle	2.11° to 6°	3.3	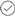 Upright lip	-
	Nasolabial angle	114.4° to 98.4°	85.2	-	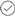 Acute
	Upper lip to Nasal Tip	114.4° to 98.4°	22.2	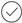	-
	Upper lip thickness	11.9 to 14.7	8.6	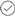 Thin	-
Hard tissue support	Mx 11 angle	60.8° to 54.8°	62.5	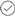 Upright	-
Mandibular Projection					
Soft tissue Projection	Pogonion	−5.2 to −1.2	−9.7	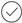	-
Hard tissue support	Md 11 angulation	67° to 61°	67.5	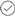 Upright	-
A-P dentoskeletal					
Intra−jaw relationship	Facial angle	167 to 173	163.2	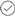 Convex	-
Maxillary	Mx 11 inclination	60.8 to 54.8	62.5	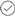 Upright	-
Mandibular	Md 11 angulation	67° to 61°	67.5	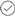 Upright	-

Note: Mx 11—The maxillary central incisor; Md 11—The mandibular central incisor.

**Table 2:** Soft tissue cephalometric analysis (STCA)—heights (NemoFab, Nemotec, Madrid, Spain).

STCA Heights					
Cephalometric Variables		Normal Range	Patient	Short	Long
Soft tissue	Interlabial gap (ULB to LLT)	1.1 to 3.3	−4	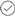 Deficient	-
	Lower lip height (LLT to Me^’^)	46.5 to 51.3	54.5	-	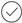
Maxillary vertical balance	Mx 11 exposure-relaxed (Mx11-ULB)	2.5 to 4.5	7	-	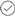 Excessive
Mandibular vertical balance	Lower lip height (LLT to Me^’^)	46.5 to 51.3	54.5	-	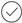

Note: Mx 11—The maxillary central incisor.

**Table 3:** Measured parameters before and after surgery (NemoFab, Nemotec, Madrid, Spain).

Parameters	Preoperative	3 Weeks Postoperative
Upper incisor (UI) exposure	7 mm	2 mm
Upper incisor to the soft tissue plane (UI-STP)	−6 mm	−8.5 mm
Maxillary occlusal plane	103 degrees	94 degrees
Mandibular occlusal plane	99 degrees	92 degrees
Articulare-Gonion-Menton (Ar-Go-Me)	136.49 degrees	128.7 degrees
The width of the mandible (Go-Go)	90.5 mm	96.2 mm

## Data Availability

Not applicable.
